# Peripheral blood immunoinflammatory biomarkers: prospective predictors of postoperative long-term survival and chronic postsurgical pain in breast cancer

**DOI:** 10.3389/fimmu.2025.1531639

**Published:** 2025-01-29

**Authors:** Baoli Li, Li Che, Huixian Li, Fangdi Min, Bolun Ai, Linxin Wu, Taihang Wang, Peixin Tan, Bingbing Fu, Jiashuo Yang, Yi Fang, Hui Zheng, Tao Yan

**Affiliations:** ^1^ Department of Anesthesiology, National Cancer Center/National Clinical Research Center for Cancer/Cancer Hospital, Chinese Academy of Medical Sciences and Peking Union Medical College, Beijing, China; ^2^ Department of Cardiology, Central Hospital of Dalian University of Technology, Dalian, China; ^3^ Department of Breast Surgical Oncology, National Cancer Center/National Clinical Research Center for Cancer/Cancer Hospital, Chinese Academy of Medical Sciences and Peking Union Medical College, Beijing, China

**Keywords:** breast cancer, disease-free survival, overall survival, chronic postsurgical pain, immunoinflammatory biomarkers

## Abstract

**Background:**

Tumor progression and chronic postsurgical pain (CPSP) in patients with breast cancer are both significantly influenced by inflammation. The associations between immunoinflammatory biomarkers and long-term survival, as well as CPSP, remain ambiguous. This study examined the predictive value of immunoinflammatory biomarkers for both long-term survival and CPSP.

**Methods:**

Data on the clinicopathological characteristics and perioperative peripheral blood immunoinflammatory biomarkers of 80 patients who underwent breast cancer surgery were retrospectively collected. Optimal cut-off values for preoperative immunoinflammatory biomarkers, including the preoperative systemic immune-inflammation index (SII), systemic inflammation response index (SIRI), neutrophil-to-lymphocyte ratio (NLR), and pan-immune-inflammation value (PIV), were established via receiver operating characteristic (ROC) curves. Kaplan−Meier curves and Cox regression analysis were used to evaluate the relationships between preoperative immunoinflammatory biomarkers and long-term survival. The relationships among the perioperative neutrophil count (NEU), monocyte count (MONO), lymphocyte count (LYM), platelet count (PLT), SII, SIRI, NLR, PIV, dynamic changes in peripheral blood cell counts, and CPSP were further assessed using logistic regression analysis.

**Results:**

Kaplan−Meier curves revealed a considerable prolongation of disease-free survival (DFS) and overall survival (OS) in the low preoperative SII, SIRI, NLR, and PIV groups. Multivariate Cox regression analysis revealed that only an elevated preoperative SIRI was an independent risk factor for postoperative DFS (HR=8.890, P=0.038). The incidence of CPSP was 28.75%. Univariate logistic regression analysis revealed that body mass index (BMI), postoperative NEU, MONO, SIRI, and PIV were negatively correlated with the occurrence of CPSP, whereas subsequent multivariate logistic regression analysis revealed that only BMI was independently associated with CPSP (OR=0.262, P=0.023).

**Conclusion:**

Elevated preoperative SIRI was an independent risk factor for poor DFS in breast cancer patients after surgery. In contrast, perioperative immunoinflammatory biomarkers had limited potential for predicting CPSP in patients who underwent breast cancer surgery.

## 1 Introduction

According to the latest data released by GLOBOCAN in 2024, breast cancer topped the list of female cancers worldwide in 2022 and significantly contributed to cancer-related mortality among women globally (1). The primary treatment strategy for breast cancer focuses on early surgical surgery, frequently accompanied by adjuvant therapies, including chemotherapy, radiotherapy, and endocrine therapy. Despite advancements in treatment, tumor recurrence and metastasis remain significant challenges for patient prognosis and long-term survival (2, 3). Consequently, identifying reliable prognostic biomarkers is crucial for optimizing treatment options and enhancing patient survival.

Inflammation is intricately associated with tumor advancement and survival (4, 5). Although protective inflammation helps the immune system eliminate stimuli and reestablish homeostasis, long-term chronic inflammation can facilitate tumor progression by stimulating tumor angiogenesis, promoting immune evasion, and causing DNA damage (6). Classic inflammatory and immune cells, such as neutrophils, monocytes, lymphocytes, and platelets, are correlated with the prognosis of numerous tumors (7). Immunoinflammatory biomarkers in peripheral blood represent the condition of the overall immunoinflammatory system in humans and have been recommended as prognostic indicators for a number of malignancies (8, 9). However, there remains insufficient support from long-term studies regarding the predictive value of immunoinflammatory biomarkers for breast cancer prognosis.

Postsurgical pain is a prevalent complication following breast cancer surgery. It has been reported that as many as 68% of patients endure moderate to severe acute pain within the first 72 hours after surgery (10). A meta-analysis indicated that almost 50% of female breast cancer patients who underwent surgery may develop chronic postsurgical pain (CPSP). Among those affected, up to 50% report moderate to severe pain, which significantly compromises their quality of life and may influence their subsequent treatments (11). Inflammation is a significant cause of acute pain, and persistent inflammation-mediated peripheral and central sensitization is an essential mechanism for facilitating the chronicity of acute postoperative pain (12–14). The relationship between perioperative immunoinflammatory biomarkers and CPSP in breast cancer patients has not been extensively studied. Hence, it is essential to explore the predictive significance and potential therapeutic applications of these markers in CPSP.

Eight commonly used inflammation indicators were included in this study, including the systemic immune-inflammation index (SII), systemic inflammation response index (SIRI), neutrophil-to-lymphocyte ratio (NLR), pan-immune-inflammation value (PIV), neutrophil count (NEU), monocyte count (MONO), lymphocyte count (LYM), and platelet count (PLT). The predictive significance of immunoinflammatory biomarkers for long-term survival and CPSP in patients who underwent breast cancer surgery with approximately 8 years of regular follow-up was assessed.

## 2 Research materials and procedures

### 2.1 Research design

This study constituted a secondary analysis of breast cancer patients who had previously participated in a prospective clinical trial and received regular follow-ups for approximately eight years. This prospective study was officially recorded in the Chictr.org.cn database on August 21, 2018 (ChiCTR1800017910). The previous enrollment was from February 2016 to February 2017 at the Cancer Hospital of the Chinese Academy of Medical Sciences. The inclusion criteria for patients were as follows (1): underwent mastectomy or breast-conserving surgery along with sentinel lymph node biopsy or axillary lymph node dissection and (2) received adjuvant therapy according to the guidelines after surgery. The exclusion criteria were as follows: (1) distant metastasis at diagnosis; (2) preoperative adjuvant radiotherapy, chemotherapy, or surgery; (3) history of other malignancies; (4) concomitant hematological or autoimmune diseases; (5) concomitant acute or chronic infections; (6) concomitant severe cardiovascular, endocrine, or neurological diseases; and (7) incomplete clinical or follow-up information.

### 2.2 Clinical data collection

Data regarding patient demographics and clinical characteristics, including age at surgery, body mass index (BMI), menstrual status, American Society of Anesthesiologists (ASA) classification, type of anesthesia, type of surgery, tumor size, type of tumor, TNM stage, histological grade, carcinoma cell embolus, nerve infiltration, estrogen receptor (ER), progesterone receptor (PR), human epidermal growth factor receptor 2 (HER2), Ki-67 expression, postoperative adjuvant therapies and other relevant clinical information, were extracted from the clinical electronic medical record system. Additionally, peripheral blood cell counts, including the NEU, MONO, LYM, and PLT, were obtained from the clinical laboratory within one week preceding and one week following surgery. The immunoinflammatory biomarkers of the patients were calculated using the following formulas: (1) SII=PLT×NEU/LYM; (2) SIRI=NEU×MONO/LYM; (3) NLR=NEU/LYM; and (4) PIV=NEU×MONO×PLT/LYM.

The preoperative SII, SIRI, NLR, and PIV were used in this study to evaluate the prognostic value of preoperative immunoinflammatory biomarkers for long-term survival, including disease-free survival (DFS) and overall survival (OS). Additionally, perioperative NEU, MONO, LYM, PLT, SII, SIRI, NLR, PIV, and dynamic changes in peripheral blood cell counts were utilized to explore the predictive significance of perioperative immunoinflammatory markers for CPSP.

### 2.3 Postoperative follow-up

The follow-up was conducted by outpatient review and telephone re-examination with a deadline of September 30, 2024. Routine follow-up occurred biannually for five years post-surgery and then annually. Recurrence or metastasis was confirmed by pathological examination following puncture/resection of the lesion or diagnostic imaging reports, including computed tomography, magnetic resonance imaging, or nuclear medicine bone scans. Information regarding the recurrence, metastasis, or death of patients was obtained from patients and their families, inpatient and outpatient records, and the local security census. CPSP was defined as (1) the emergence of postsurgical pain persisting for ≥3 months; (2) pain localized to or associated with the area of surgery, including the chest wall, axilla, or upper limb on the surgical side; and (3) the exclusion of alternate etiologies for the pain, including infection, tumor recurrence, and preexisting pain conditions (15).

### 2.4 Statistical analysis

SPSS 27.0 and GraphPad Prism 10.0 were used to conduct the statistical evaluations. To describe the data, quantitative parameters adhering to a normal distribution are presented as the means ± standard deviations (SDs), those deviating from a normal distribution are presented as the medians (P25, P75), and categorical variables are presented as numbers (proportions). For group comparisons, continuous variables following a normal distribution were analyzed using the Student’s t-test, deviations from this norm were assessed via the Mann−Whitney U test, and qualitative variables were examined via Pearson’s chi-square test or Fisher’s exact test. The optimal cut-off values of preoperative immunoinflammatory biomarkers were identified by receiver operating characteristic (ROC) curves. Kaplan−Meier curves and log-rank tests were employed to evaluate the associations between immunoinflammatory biomarkers and long-term survival. Prognostic risk factors for breast cancer patients were ascertained through univariate and multivariate Cox regression analysis, whereas factors influencing CPSP were identified via univariate and multivariate logistic regression analysis. P <0.05 was considered a sign of a notable statistical discrepancy.

## 3 Results

### 3.1 Clinicopathological features

**Figure 1:**
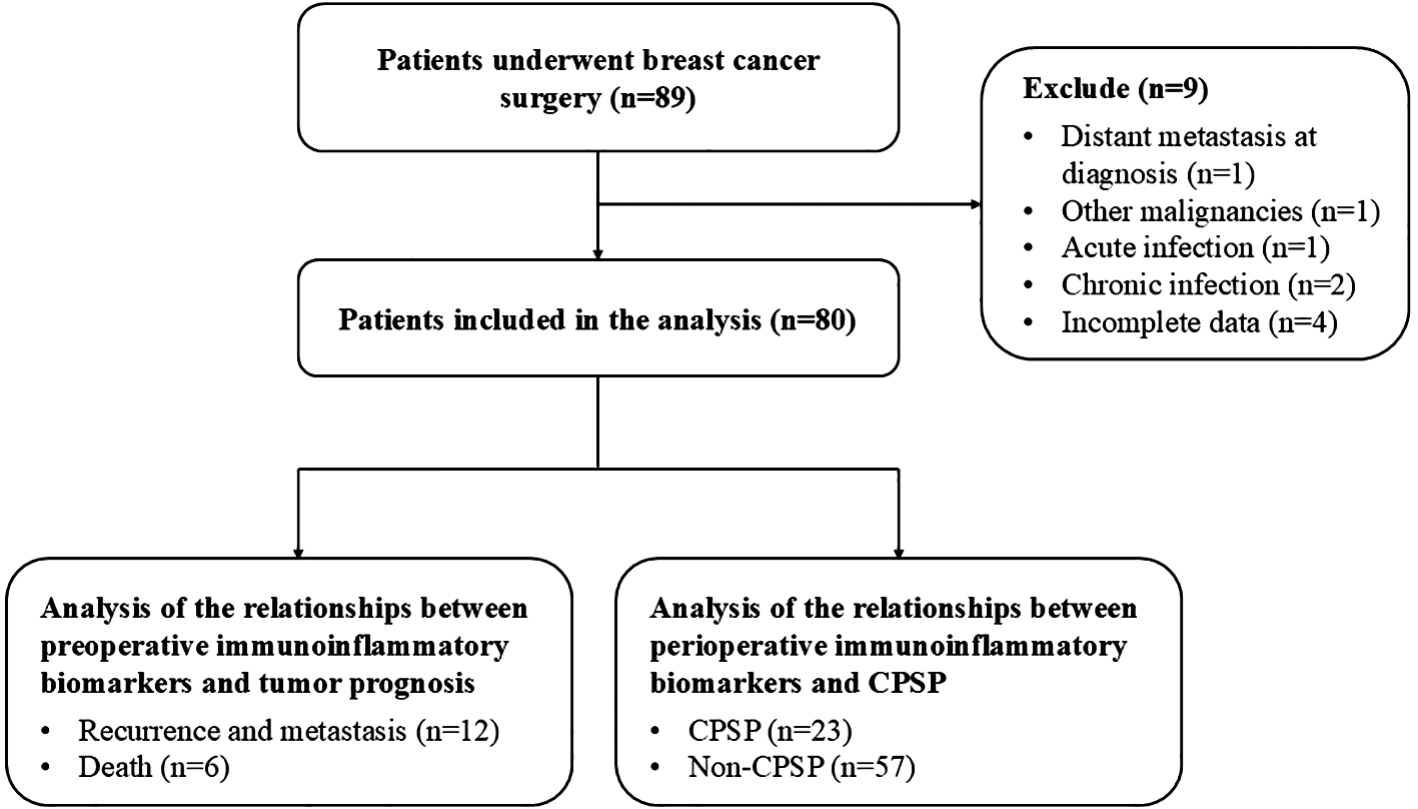
Flowchart of the study cohort. CPSP, chronic postsurgical pain.

The study included 80 female breast cancer patients who satisfied the selection criteria, as shown in the flowchart (**Figure 1**). The clinicopathological properties of the participants are presented in **Table 1**. Forty patients were under 50 years of age, 41 had a BMI below 22 kg/m², and 37 were postmenopausal. Among the patients, 48 were ASA I, 26 were ASA II, and 6 were ASA III. The surgery types included breast-conserving surgery (31 patients) and mastectomy (49 patients). The anesthesia types included total intravenous anesthesia (TIVA) for 42 patients and combined intravenous–inhalation anesthesia (CIVIA) for 38 patients. Among the tumor classifications, 3 cases were identified as carcinoma *in situ*, with the remaining 77 categorized as invasive. With respect to TNM staging, 29 patients were in stages Tis and I, whereas 51 patients were classified into stages II and III. Histological grades were distributed as follows: 9 patients were classified as grade I, 45 as grade II, and 26 as grade III. Evidence of carcinoma cell embolus was present in 24 patients, nerve infiltration in 10 patients, and lymph node metastasis in 28 patients. In terms of receptor status, 59 patients were ER-positive (ER+), 57 were PR-positive (PR+), and 17 were HER2-positive (HER2+). Postoperative interventions included chemotherapy for 63 patients, radiotherapy for 35 patients, endocrine therapy for 59 patients, and targeted therapy for 17 patients.

**Table 1:** Clinicopathological features of eighty patients.

Characteristic	Number	Percentage (%)	Characteristic	Number	Percentage (%)
Total	80	100	Lymph node metastasis		
Age	49.73 ± 10.70		No	52	65.0
< 50 years	40	50.0	Yes	28	35.0
≥ 50 years	40	50.0	ER		
BMI	21.67 (20.92,22.63)		–	21	26.2
< 22 kg/m^2^	41	51.2	+	59	73.8
≥ 22 kg/m^2^	39	48.8	PR		
Menopausal			–	23	28.7
Premenopausal	43	53.8	+	57	71.3
Postmenopausal	37	46.2	HER2		
ASA classification			–	63	78.8
I	48	60.0	+	17	21.2
II	26	32.5	Ki-67		
III	6	7.5	≤ 20%	33	41.2
Anesthesia			> 20%	47	58.8
CIVIA	38	47.5	Postoperative chemotherapy		
TIVA	42	52.5	No	17	21.2
Surgery			Yes	63	78.8
Mastectomy	49	61.2	Postoperative radiotherapy		
Breast conserving surgery	31	38.8	No	45	56.2
Tumor size			Yes	35	43.8
< 2 cm	37	46.2	Endocrine therapy		
≥ 2 cm	43	53.8	No	21	26.2
Tumor type			Yes	59	73.8
Carcinoma in situ	3	3.8	Targeted therapy		
Invasive carcinoma	77	96.2	No	63	78.8
TNM stage			Yes	17	21.2
Tis + I	29	36.3	Recurrence/Metastasis		
II + III	51	63.7	No	68	85.0
Histological grade			Yes	12	15.0
I	9	11.2	Death		
II	45	56.3	No	74	92.5
III	26	32.5	Yes	6	7.5
Carcinoma cell embolus			CPSP		
No	56	70.0	No	57	71.3
Yes	24	30.0	Yes	23	28.7
Nerve infiltration					
No	70	87.5			
Yes	10	12.5			

BMI, body mass index; ASA, American Society of Anesthesiologists; CIVIA, combined intravenous–inhalation anesthesia; TIVA, total intravenous anesthesia; TNM, tumor node metastasis; ER, estrogen receptor; PR, progesterone receptor; HER2, human epidermal growth factor receptor 2; CPSP, chronic postsurgical pain. Continuous variables are summarized as the means ± SDs or medians (P25, P75). Categorical variables are summarized as the number of subjects and percentage.

### 3.2 Optimal cut-off values of preoperative immunoinflammatory biomarkers

**Figure 2:**
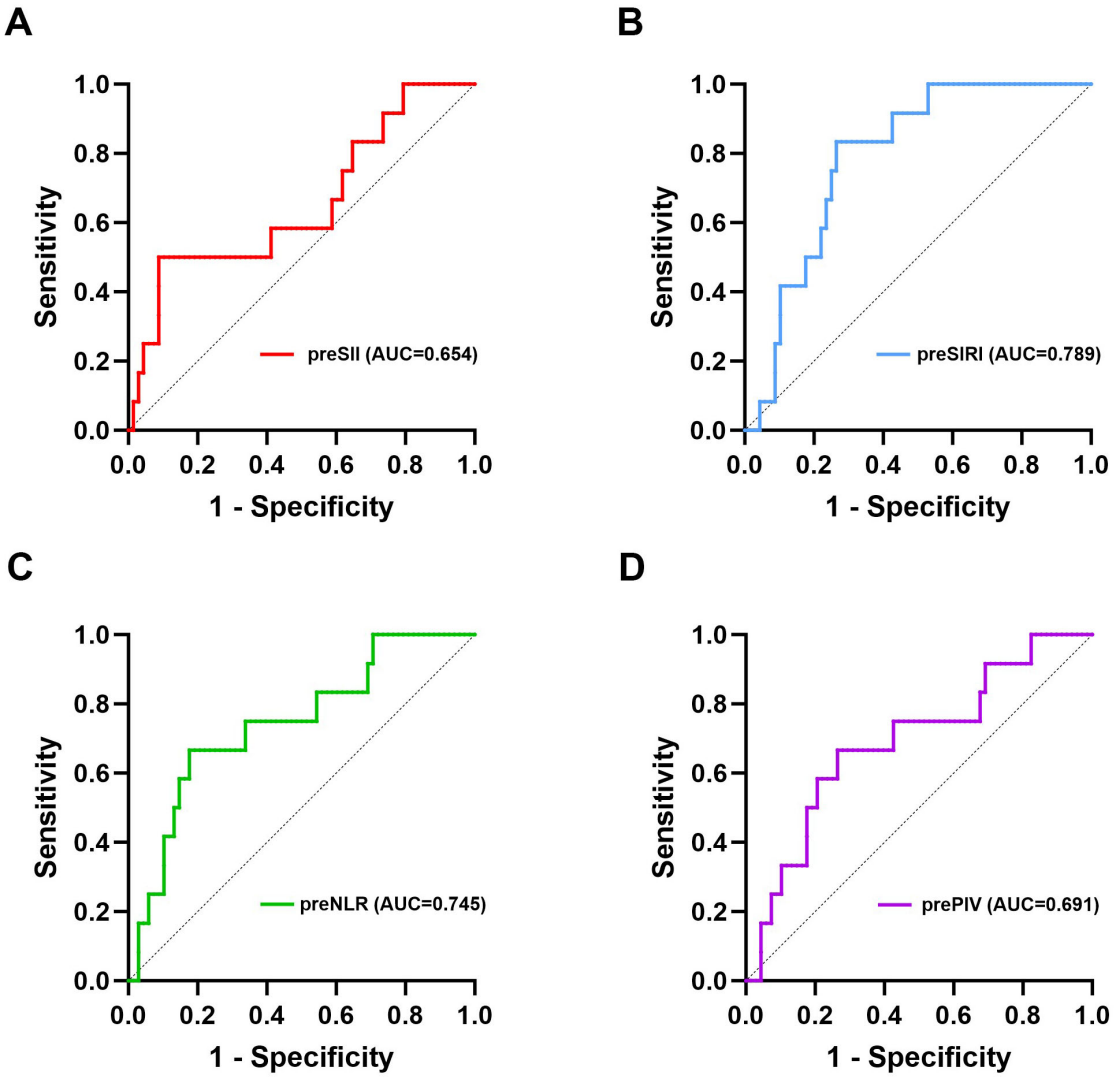
ROC curves of the preoperative SII, SIRI, NLR, and PIV for predicting long-term survival. **(A)** ROC curve of the preoperative SII for DFS; **(B)** ROC curve of the preoperative SIRI for DFS; **(C)** ROC curve of the preoperative NLR for DFS; **(D)** ROC curve of the preoperative PIV for DFS. pre, preoperative; SII, systemic immune-inflammation index; SIRI, systemic inflammation response index; NLR, neutrophil-to-lymphocyte ratio; PIV, pan-immune-inflammation value; DFS, disease-free survival.

Given the low mortality at the end of the follow-up, which was also affected by causes of death unrelated to tumor progression, we constructed ROC curves on the basis of recurrence and metastasis, as illustrated in **Figure 2**. The optimal cut-off values for the preoperative SII, SIRI, NLR, and PIV, ascertained through the maximal Youden’s index, were 757.00, 0.79, 2.50, and 172.33, respectively, as presented in **Table 2**. Patients were subsequently categorized into two corresponding groups according to these cut-off values: for the SII, a low group (<757.00, N=68) and a high group (≥757.00, N=12); for the SIRI, a low group (<0.79, N=52) and a high group (≥0.79, N=28); for the NLR, a low group (<2.50, N=60) and a high group (≥2.50, N=20); and for the PIV, a low group (<172.33, N=54) and a high group (≥172.33, N=26).

**Table 2:** The cut-off values of the preoperative SII, SIRI, NLR, and PIV for predicting long-term survival.

Variables	AUC	95% CI	Sensitivity	Specificity	Youden’s index	Cut-off value
pre SII	0.654	0.474-0.835	0.500	0.912	0.412	757.00
pre SIRI	0.789	0.681-0.897	0.833	0.735	0.568	0.79
pre NLR	0.745	0.595-0.896	0.667	0.824	0.491	2.50
pre PIV	0.691	0.527-0.855	0.667	0.735	0.402	172.33

AUC, area under the curve; CI, confidence interval; pre, preoperative; SII, systemic immune-inflammation index; SIRI, systemic inflammation response index; NLR, neutrophil-to-lymphocyte ratio; PIV, pan-immune-inflammation value.

### 3.3 Correlations between preoperative immunoinflammatory biomarkers and clinicopathological characteristics

The correlations between preoperative immunoinflammatory biomarkers and clinicopathological characteristics are shown in **Table 3**. The chi-square test revealed that patients classified in the high preoperative NLR group were substantially younger at the time of surgery (P=0.009) and presented a greater prevalence of premenopausal status (P=0.007). Additionally, a significant decrease in the incidence of HER2+ breast cancer patients was observed in the high preoperative PIV group compared to the low PIV group (P=0.008). No statistically significant differences were identified between the high and low preoperative SII groups regarding any clinicopathological characteristics, a finding that was consistent across the high and low preoperative SIRI groups.

**Table 3:** Relationships between the preoperative SII, SIRI, NLR, and PIV and clinicopathological characteristics.

Characteristic	pre SII			pre SIRI			pre NLR			pre PIV		
	Low (%)	High (%)	*p*	Low (%)	High (%)	*p*	Low (%)	High (%)	*p*	Low (%)	High (%)	*p*
**Total**	68	12		52	28		60	20		54	26	
**Age (years)**	50.15 ± 10.74	47.33 ± 10.59	0.404	50.38 ± 10.79	48.50 ± 10.62	0.456	51.50 ± 10.38	44.40 ± 10.08	**0.009**	50.06 ± 10.95	49.04 ± 10.34	0.693
**BMI**			0.178			0.761			0.897			0.747
< 22 kg/m^2^	37(54.4)	4(33.3)		26(50.0)	15(53.6)		31(51.7)	10(50.0)		27(50.0)	14(53.8)	
≥ 22 kg/m^2^	31(45.6)	8(66.7)		26(50.0)	13(46.4)		29(48.3)	10(50.0)		27(50.0)	12(46.2)	
**Menopausal**			0.109			0.063			**0.007**			0.148
Premenopausal	34(50.0)	9(75.0)		24(46.2)	19(67.9)		27(45.0)	16(80.0)		26(48.1)	17(65.4)	
Postmenopausal	34(50.0)	3(25.0)		28(53.8)	9(32.1)		33(55.0)	4(20.0)		28(51.9)	9(34.6)	
**ASA classification**			1.000			1.000			0.656			0.931
I	41(60.3)	7(58.3)		31(59.6)	17(60.7)		34(56.7)	14(70.0)		33(61.1)	15(57.7)	
II	22(32.4)	4(33.3)		17(32.7)	9(32.1)		21(35.0)	5(25.0)		17(31.5)	9(34.6)	
III	5(7.3)	1(8.4)		4(7.7)	2(7.2)		5(8.3)	1(5.0)		4(7.4)	2(7.7)	
**Anesthesia**			0.661			0.121			0.438			0.579
CIVIA	33(48.5)	5(41.7)		28(53.8)	10(35.7)		30(50.0)	8(40.0)		27(50.0)	11(42.3)	
TIVA	35(51.5)	7(58.3)		24(46.2)	18(64.3)		30(50.0)	12(60.0)		27(50.0)	15(57.7)	
**Surgery**			1.000			0.683			0.508			0.971
Mastectomy	42(61.8)	7(58.3)		31(59.6)	18(64.3)		38(63.3)	11(55.0)		33(61.1)	16(61.5)	
Breast conserving surgery	26(38.2)	5(41.7)		21(40.4)	10(35.7)		22(36.7)	9(45.0)		21(38.9)	10(38.5)	
**Tumor size**			0.330			0.655			0.244			0.641
< 2 cm	33(48.5)	4(33.3)		25(48.1)	12(42.9)		30(50.0)	7(35.0)		24(44.4)	13(50.0)	
≥ 2 cm	35(51.5)	8(66.7)		27(51.9)	16(57.1)		30(50.0)	13(65.0)		30(55.6)	13(50.0)	
**Tumor type**			0.390			1.000			1.000			1.000
Carcinoma in situ	2(2.9)	1(8.3)		2(3.8)	1(3.6)		2(3.3)	1(5.0)		2(96.3)	1(3.8)	
Invasive carcinoma	66(97.1)	11(91.7)		50(96.2)	27(96.4)		58(96.7)	19(95.0)		52(3.7)	25(96.2)	
**TNM stage**			0.922			0.575			0.893			0.833
Tis + I	24(35.3)	5(41.7)		20(38.5)	9(32.1)		22(36.7)	7(35.0)		20(37.0)	9(34.6)	
II + III	44(64.7)	7(58.3)		32(61.5)	19(67.9)		38(63.3)	13(65.0)		34(63.0)	17(65.4)	
**Histological grade**			0.356			0.588			0.368			0.941
I	7(10.3)	2(16.7)		7(13.5)	2(7.1)		6(10.0)	3(15.0)		6(11.1)	3(11.5)	
II	37(54.4)	8(66.6)		27(51.9)	18(64.3)		32(53.3)	13(65.0)		31(57.4)	14(53.8)	
III	24(35.3)	2(16.7)		18(34.6)	8(28.6)		22(36.7)	4(20.0)		17(31.5)	9(34.7)	
**Carcinoma cell embolus**			0.539			0.413			0.091			0.917
No	49(72.1)	7(58.3)		38(73.1)	18(64.3)		45(75.0)	11(55.0)		38(70.4)	18(69.2)	
Yes	19(27.9)	5(41.7)		14(26.9)	10(35.7)		15(25.0)	9(45.0)		16(29.6)	8(30.8)	
**Nerve infiltration**			0.058			1.000			0.435			0.367
No	62(91.2)	8(66.7)		46(88.5)	24(85.7)		54(90.0)	16(80.0)		49(90.7)	21(80.8)	
Yes	6(8.8)	4(33.3)		6(11.5)	4(14.3)		6(10.0)	4(20.0)		5(9.3)	5(19.2)	
**Lymph node metastasis**			0.646			0.694			1.000			0.293
No	43(63.2)	9(75.0)		33(63.5)	19(67.9)		39(65.0)	13(65.0)		33(61.1)	19(73.1)	
Yes	25(36.8)	3(25.0)		19(36.5)	9(32.1)		21(35.0)	7(35.0)		21(38.9)	7(26.9)	
**ER**			0.240			0.211			0.187			0.654
–	20(29.4)	1(8.3)		16(30.8)	5(17.9)		18(30.0)	3(15.0)		15(27.8)	6(23.1)	
+	48(70.6)	11(91.7)		36(69.2)	23(82.1)		42(70.0)	17(85.0)		39(72.2)	20(76.9)	
**PR**			0.177			0.114			0.117			0.437
–	22(32.4)	1(8.3)		18(34.6)	5(17.9)		20(33.3)	3(15.0)		17(31.5)	6(23.1)	
+	46(67.6)	11(91.7)		34(65.4)	23(82.1)		40(66.7)	17(85.0)		37(68.5)	20(76.9)	
**HER2**			0.422			0.264			1.000			**0.008**
–	52(76.5)	11(91.7)		39(75.0)	24(85.7)		47(78.3)	16(80.0)		38(70.4)	25(96.2)	
+	16(23.5)	1(8.3)		13(25.0)	4(14.3)		13(21.7)	4(20.0)		16(29.6)	1(3.8)	
**Ki-67**			0.775			0.091			0.088			0.725
≤ 20%	29(42.6)	4(33.3)		25(48.1)	8(28.6)		28(46.7)	5(25.0)		23(42.6)	10(38.5)	
> 20%	39(57.4)	8(66.7)		27(51.9)	20(71.4)		32(53.3)	15(75.0)		31(57.4)	16(61.5)	

pre, preoperative; SII, systemic immune-inflammation index; SIRI, systemic inflammation response index; NLR, neutrophil-to-lymphocyte ratio; PIV, pan-immune-inflammation value; BMI, body mass index; ASA, American Society of Anesthesiologists; CIVIA, combined intravenous–inhalation anesthesia; TIVA, total intravenous anesthesia; TNM, tumor node metastasis; ER, estrogen receptor; PR, progesterone receptor; HER2, human epidermal growth factor receptor 2. Continuous variables are summarized as the means ± SDs or medians (P25, P75). Categorical variables are summarized as the number of subjects and percentage. The values in bold represent P <0.05.

### 3.4 Survival analysis

**Figure 3:**
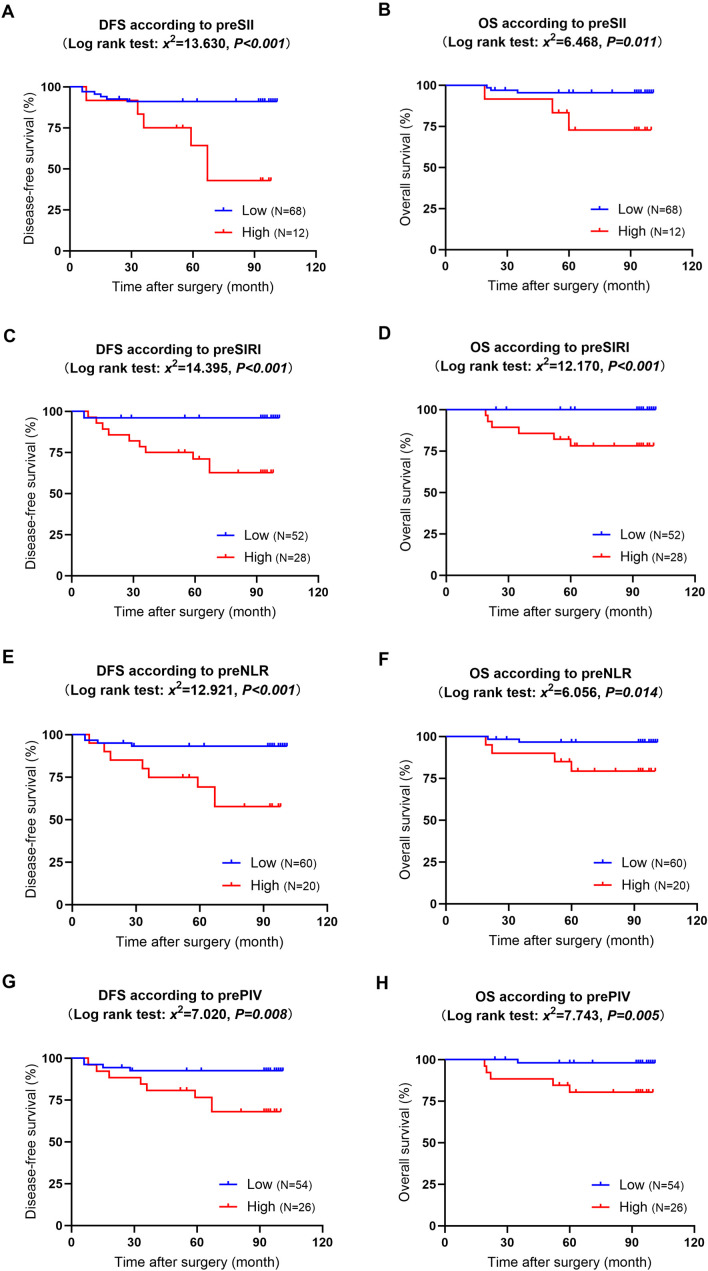
Kaplan−Meier curves and log-rank tests for the relationships between preoperative immunoinflammatory biomarkers and long-term survival. **(A)**: DFS in patients with high versus low preoperative SII; **(B)**: OS in patients with high versus low preoperative SII; **(C)**: DFS in patients with high versus low preoperative SIRI; **(D)**: OS in patients with high versus low preoperative SIRI; **(E)**: DFS in patients with high versus low preoperative NLR; **(F)**: OS in patients with high versus low preoperative NLR; **(G)**: DFS in patients with high versus low preoperative PIV;**(H)**: OS in patients with high versus low preoperative PIV. DFS, disease-free survival; OS, overall survival; pre, preoperative; SII, systemic immune-inflammation index; SIRI, systemic inflammation response index; NLR, neutrophil-to-lymphocyte ratio; PIV, pan-immune-inflammation value.

The median follow-up time was 94 months, ranging from 19 to 101 months. During the follow-up, 12 patients experienced recurrence or metastasis, and six patients died. The Kaplan−Meier curves indicated that elevated preoperative SII, SIRI, NLR, and PIV values were significantly associated with poor DFS and OS (P<0.05) (refer to **Figure 3**). **Supplementary Figure 1** shows the Kaplan−Meier curves stratified by the clinicopathologic features of the patients. The results indicated that patients with high Ki-67 expression and those with positive carcinoma cell embolus had significantly shorter DFS than their counterparts with low Ki-67 expression and negative carcinoma cell embolus (P=0.012 and P=0.017, respectively). While not statistically significant, there was a trend toward decreased OS in patients with axillary sentinel lymph node metastasis (P=0.061). Analysis of additional clinicopathologic variables through Kaplan−Meier curves and log-rank tests did not reveal any statistically significant differences.

### 3.5 Factors affecting long-term prognosis

Cox regression analysis was conducted to explore the factors influencing DFS and OS (refer to **Tables 4**, **5**). Univariate Cox regression analysis indicated that positive carcinoma cell embolus (HR=3.677, P=0.026) and high Ki-67 expression (HR=8.820, P=0.037) were related factors for DFS. Additionally, the preoperative SII (HR=6.437, P=0.001), preoperative SIRI (HR=10.574, P=0.002), preoperative NLR (HR=6.712, P=0.002), and preoperative PIV (HR=4.398, P=0.016) were negatively associated with DFS. Notably, multivariate Cox regression analysis revealed that only an elevated preoperative SIRI was an independent risk factor for decreased DFS (HR=8.890, P=0.038). In terms of OS, univariate Cox regression analysis indicated that the preoperative SII (HR=6.168, P=0.026), preoperative NLR (HR=6.393, P=0.032), and preoperative PIV (HR=11.215, P=0.027) were negatively associated with OS. However, multivariate Cox regression analysis revealed that none of these variables independently predicted OS.

**Table 4:** Univariate and multivariate Cox regression analysis for DFS.

Characteristic	Univariate analysis			Multivariate analysis		
	HR	95% CI	*P*	HR	95% CI	*P*
Carcinoma cell embolus						
No	1			1		
Yes	3.677	1.166-11.595	**0.026**	3.203	0.980-10.466	0.054
Ki-67						
≤ 20%	1			1		
> 20%	8.820	1.138-68.355	**0.037**	6.693	0.826-54.220	0.075
pre SII						
< 757.00	1			1		
≥ 757.00	6.437	2.064-20.077	**0.001**	2.552	0.435-14.969	0.299
pre SIRI						
< 0.79	1			1		
≥ 0.79	10.574	2.312-48.370	**0.002**	8.890	1.123-70.350	**0.038**
pre NLR						
< 2.50	1			1		
≥ 2.50	6.712	2.016-22.346	**0.002**	0.847	0.111-6.487	0.873
pre PIV						
< 172.33	1			1		
≥ 172.33	4.398	1.323-14.617	**0.016**	0.565	0.073-4.375	0.585

HR, hazard ratio; CI, confidence interval; SII, systemic immune-inflammation index; SIRI, systemic inflammation response index; NLR, neutrophil-to-lymphocyte ratio; PIV, pan-immune-inflammation value. The values in bold represent P <0.05.

**Table 5:** Univariate and multivariate Cox regression analysis for OS.

Characteristic	Univariate analysis			Multivariate analysis		
	HR	95% CI	*P*	HR	95% CI	*P*
pre SII						
< 757.00	1			1		
≥ 757.00	6.168	1.242-30.628	**0.026**	1.149	0.116-11.386	0.905
pre NLR						
< 2.50	1			1		
≥ 2.50	6.393	1.170-34.932	**0.032**	2.014	0.164-24.678	0.584
pre PIV						
< 172.33	1			1		
≥ 172.33	11.215	1.310-96.039	**0.027**	6.928	0.566-84.845	0.130

HR, hazard ratio; CI, confidence interval; SII, systemic immune-inflammation index; NLR, neutrophil-to-lymphocyte ratio; PIV, pan-immune-inflammation value. The values in bold represent P <0.05.

### 3.6 Characteristics of patients with and without CPSP

The clinicopathological characteristics associated with the presence or absence of CPSP are summarized in **Table 6**. The incidence of CPSP was 28.75%. The chi-square test results indicated that the CPSP group presented a greater percentage of patients with a BMI <22 kg/m² (P=0.010). Patients in the CPSP group demonstrated lower levels of postoperative NEU (P=0.004), postoperative MONO (P=0.014), and postoperative SIRI (P=0.021) than those in the non-CPSP group. Additionally, as indicated in **Supplementary Table 1**, we conducted an analysis of the relationship between CPSP and dynamic changes in peripheral blood cell counts, including the differences and ratios between the postoperative and preoperative levels of each cell type. The results indicated that patients in the CPSP group exhibited a smaller change in NEU (calculated as the postoperative NEU minus the preoperative NEU) (P=0.026). Other characteristics, such as age at surgery, menstrual status, type of anesthesia, type of surgery, and preoperative immunoinflammatory markers, did not significantly correlate with the occurrence of CPSP.

**Table 6:** Characteristics of patients with and without CPSP.

Characteristic	Non-CPSP (N=57)	CPSP (N=23)	*P*	Characteristic	Non-CPSP (N=57)	CPSP (N=23)	*P*
**Age (years)**	48.77 ± 10.64	52.09 ± 10.72	0.212	**Nerve infiltration**			0.076
**BMI**			**0.010**	No	47(82.5)	23(100.0)	
< 22 kg/m^2^	24(42.1)	17(73.9)		Yes	10(17.5)	0(0.0)	
≥ 22 kg/m^2^	33(57.9)	6(26.1)		**Lymph node metastasis**			0.979
**Menopausal**			0.500	No	37(64.9)	15(65.2)	
Premenopausal	32(56.1)	11(47.8)		Yes	20(35.1)	8(34.8)	
Postmenopausal	25(43.9)	12(52.2)		**Postoperative chemotherapy**			0.815
**ASA classification**			0.627	No	13(22.8)	4(17.4)	
I	36(63.2)	12(52.2)		Yes	44(77.2)	19(82.6)	
II	17(29.8)	9(39.1)		**Postoperative radiotherapy**			0.975
III	4(7.0)	2(8.7)		No	32(56.1)	13(56.5)	
**Anesthesia**			0.595	Yes	25(43.9)	10(43.5)	
CIVIA	26(45.6)	12(52.2)		**Endocrine therapy**			0.983
TIVA	31(54.4)	11(47.8)		No	15(26.3)	6(26.1)	
**Surgery**			0.117	Yes	42(73.7)	17(73.9)	
Mastectomy	38(66.7)	11(47.8)		**Targeted therapy**			0.330
Breast conserving surgery	19(33.3)	12(52.2)		No	47(82.5)	16(69.6)	
**Tumor size**			0.096	Yes	10(17.5)	7(30.4)	
< 2 cm	23(40.4)	14(60.9)		**pre NEU (×10^9^/L)**	3.40(3.09,4.17)	3.08(2.82,3.73)	0.088
≥ 2 cm	34(59.6)	9(39.1)		**pre MONO (×10^9^/L)**	0.39(0.30,0.49)	0.34(0.30,0.43)	0.292
**Tumor type**			1.000	**pre LYM (×10^9^/L)**	1.82(1.36,2.33)	1.78(1.61,2.33)	0.774
Carcinoma in situ	2(3.5)	1(4.3)		**pre PLT (×10^9^/L)**	235.00(203.00,267.00)	240.00(218.50,266.50)	0.636
Invasive carcinoma	55(96.5)	22(95.7)		**pre SII**	437.88(278.78,667.43)	416.43(309.37,509.02)	0.675
**TNM stage**			0.393	**pre SIRI**	0.70(0.49,1.14)	0.60(0.41,0.74)	0.169
Tis + I	19(33.3)	10(43.5)		**pre NLR**	2.04(1.34,2.71)	1.72(1.32,2.14)	0.197
II + III	38(66.7)	13(56.5)		**pre PIV**	153.86(105.07,249.26)	154.08(120.81,165.04)	0.473
**Histological grade**			0.329	**post NEU (×10^9^/L)**	7.50(5.76,8.88)	6.04(5.08,6.89)	**0.004**
I	6(10.5)	3(13.0)		**post MONO (×10^9^/L)**	0.63(0.49,0.71)	0.48(0.41,0.55)	**0.014**
II	35(61.4)	10(43.5)		**post LYM (×10^9^/L)**	0.88(0.62,1.35)	0.93(0.66,1.31)	0.898
III	16(28.1)	10(43.5)		**post PLT (×10^9^/L)**	227.00(181.00,262.00)	236.00(210.00,255.00)	0.702
**Carcinoma cell embolus**			0.628	**post SII**	1808.74(1095.64,2479.03)	1378.67(1138.47,2175.72)	0.262
No	39(68.4)	17(73.9)		**post SIRI**	4.51(3.24,8.15)	3.50(2.34,4.64)	**0.021**
Yes	18(31.6)	6(26.1)		**post NLR**	8.41(5.36,11.90)	6.53(4.41,9.20)	0.138
				**post PIV**	993.55((598.17,1777.21)	912.47(549.45,1063.33)	0.055

CPSP, chronic postsurgical pain; BMI, body mass index; ASA, American Society of Anesthesiologists; CIVIA, combined intravenous–inhalation anesthesia; TIVA, total intravenous anesthesia; TNM, tumor node metastasis; ER, estrogen receptor; PR, progesterone receptor; HER2, human epidermal growth factor receptor 2; pre, preoperative; post, postoperative; NEU, neutrophil count; MONO, monocyte count; LYM, lymphocyte count; PLT, platelet count; SII, systemic immune-inflammation index; SIRI, systemic inflammation response index; NLR, neutrophil-to-lymphocyte ratio; PIV, pan-immune-inflammation value. Continuous variables are summarized as the means ± SDs or medians (P25, P75). Categorical variables are summarized as the number of subjects and percentage. The values in bold represent P <0.05.

### 3.7 Factors affecting CPSP

This study investigated the factors affecting CPSP using logistic regression models (refer to **Table 7**). Univariate logistic regression analysis revealed several factors associated with CPSP, including BMI (OR=0.257, P=0.013), postoperative NEU (OR=0.739, P=0.013), postoperative MONO (OR=0.025, P=0.020), postoperative SIRI (OR=0.757, P=0.017), and postoperative PIV (OR=0.999, P=0.035). However, dynamic changes in any peripheral blood cell count were not found to be correlated with CPSP, as detailed in **Supplementary Table 2**. Although not statistically significant, the change in NEU (calculated as the postoperative NEU minus the preoperative NEU) appeared to influence CPSP (P=0.051). According to the results of multivariate logistic regression analysis, BMI (OR=0.262, P=0.023) was independently associated with CPSP. Specifically, patients with a BMI <22 kg/m^2^ were found to be at a greater risk of developing CPSP than those with a BMI ≥22 kg/m^2^.

**Table 7:** Univariate and multivariate logistic regression analysis for CPSP.

Characteristic	Univariate analysis			Multivariate analysis		
	OR	95% CI	*P*	OR	95% CI	*P*
BMI						
< 22 kg/m^2^	1			1		
≥ 22 kg/m^2^	0.257	0.088-0.748	**0.013**	0.262	0.082-0.833	**0.023**
**post NEU (×10^9^/L)**	0.739	0.582-0.938	**0.013**	0.823	0.599-1.130	0.228
**post MONO (×10^9^/L)**	0.025	0.001-0.562	**0.020**	0.146	0.004-5.537	0.299
**post SIRI**	0.757	0.602-0.952	**0.017**	0.762	0.444-1.307	0.324
**post PIV**	0.999	0.998-1.000	**0.035**	1.001	0.999-1.003	0.495

CPSP, chronic postsurgical pain; OR, odds ratio; CI, confidence interval; BMI, body mass index; post, postoperative; NEU, neutrophil count; MONO, monocyte count; SIRI, systemic inflammation response index; PIV, pan-immune-inflammation value. The values in bold represent P <0.05.

## 4 Discussion

Breast cancer remains a leading cause of morbidity and mortality among women globally. Established prognostic biomarkers, such as TNM stage, histologic grade, and molecular markers in tumor pathology, including ER, PR, HER2, and Ki-67, have received extensive clinical attention. However, the complexity of testing methodologies, high costs, and time demands considerably limit their clinical applications, particularly for patients diagnosed with early-stage breast cancer (16–18).

Inflammation plays a vital role in tumor progression (19, 20). Chronic inflammation has been shown to accelerate tumor proliferation, facilitate angiogenesis, and promote tumor metastasis, all of which are closely associated with the prognosis of breast cancer patients (21). Notably, peripheral blood immunoinflammatory biomarkers can not only reflect systemic immunoinflammatory conditions but also provide advantages in terms of simplicity, cost-effectiveness, and dependability, making them valuable for predicting survival in breast cancer patients. Nevertheless, the specific relationships between these immunoinflammatory biomarkers and the long-term survival of breast cancer patients remain largely unexplored.

This study investigated the predictive significance of preoperative immunoinflammatory biomarkers for the long-term survival of patients who participated in a previous clinical trial and underwent breast cancer surgery, with regular follow-ups lasting approximately eight years. The Kaplan−Meier curves indicated that patients with elevated preoperative NLR, SII, SIRI, and PIV values had significantly decreased DFS and OS. This finding aligns with the literature, suggesting that elevated immunoinflammatory biomarkers are associated with poorer survival across various malignancies (22–30).

Recent studies have reported that the NLR is a more reliable marker of systemic immunological status than inflammatory cell counts alone, effectively predicting outcomes in various solid tumors, especially breast cancer (31). A meta-analysis encompassing 31 studies explored the relationship between the preoperative NLR and the prognosis of operable breast cancer patients, confirming a significant association between an elevated preoperative NLR and increased rates of ER+ tumors, as well as shorter DFS and OS (8). Two clinical trials focusing on Asian breast cancer patients, which used NLR cut-off values of 2.57 and 2.50, corroborated the finding that an elevated NLR correlates with a worse prognosis, even in patients with triple-negative and luminal A breast cancer subtypes (32, 33). The SII, derived from peripheral blood neutrophils, platelets, and lymphocytes, provides a comprehensive overview of immunological and inflammatory conditions. A meta-analysis involving 2,642 breast cancer patients from eight studies demonstrated that those with an elevated SII experienced poorer survival (34). According to a study utilizing 758.0 as a cut-off value, a lower SII was linked to improved survival in patients with HER2+ metastatic breast cancer receiving chemotherapy in combination with trastuzumab (35). The SIRI, which integrates peripheral blood monocyte, neutrophil, and lymphocyte counts, has emerged as a robust prognostic marker associated with adverse outcomes across multiple malignancies. Zhu et al. demonstrated that the SIRI serves as an independent prognostic factor for breast cancer patients, with an optimal cut-off value of 0.80, which closely matches the cut-off value of 0.79 established in this study (36). A meta-analysis encompassing eight trials with a total of 2997 patients confirmed that an elevated SIRI is associated with a larger tumor size, more advanced stages, and worse OS (37). The PIV has been recognized as a promising prognostic biomarker across various malignancies based on combinations of peripheral blood neutrophils, monocytes, platelets, and lymphocytes (38). Previous studies have indicated that breast cancer patients with lower preoperative PIV tend to show better responses to neoadjuvant chemotherapy and experience longer DFS and OS (39, 40). Although variability exists in PIV cut-off values across studies, recent investigations have consistently supported its prognostic relevance in breast cancer (41–43). This variability may be attributable to the early stage of PIV research and the small number of studies.

Notably, the univariate Cox regression analysis conducted in this study identified several predictors of DFS, including both preoperative immunoinflammatory markers and tumor characteristics such as carcinoma cell emboli and Ki-67 expression. These findings underscore the importance of breast cancer tumor pathology in prognostic assessments. According to the multivariate Cox regression analysis, compared to the other three immunoinflammatory biomarkers, only an elevated preoperative SIRI emerged as an independent risk factor for decreased DFS. This finding aligns with previous research that indicated the lack of independence of the preoperative NLR as a prognostic biomarker, particularly in specific subtypes, such as ER+ HER2- early breast cancer, where other clinical prognostic factors may exert a significant influence (44, 45). In conclusion, identifying preoperative immunoinflammatory biomarkers as predictive tools facilitates the improvement of patient stratification and management strategies. For example, clinicians may consider adopting a more aggressive adjuvant treatment and follow-up strategy for patients exhibiting elevated preoperative SIRI, potentially involving early comprehensive adjuvant therapy, enhanced recurrence surveillance, or additional imaging.

This study further investigated the correlation between perioperative immunoinflammatory markers and CPSP. Univariate logistic regression analysis demonstrated that postoperative NEU, MONO, SIRI, and PIV were significantly correlated with CPSP, indicating that these immunoinflammatory markers may reflect the underlying inflammatory processes contributing to chronic pain. The current literature theoretically supports this observation by highlighting the significant role of inflammation in postoperative pain. The influence of inflammation on acute pain is attributed primarily to the extensive activation of immunoinflammatory cells and the subsequent release of several proinflammatory cytokines induced by surgical tissue injury (12, 46). The continuous release of inflammatory mediators progressively activates microglia, increasing neuronal sensitivity and ultimately leading to central sensitization (47, 48). Notable inflammatory mediators, including prostaglandins and substance P, intensify nociceptive signaling and substantially affect the modulation of both central and peripheral pain pathways (49, 50). Consequently, the peripheral and central sensitization induced by chronic inflammation collectively contributes to the transition from acute postoperative pain to CPSP (13, 14).

The interaction between immunoinflammatory cells and the central nervous system in the modulation of pain underscores the importance of further exploration of the connections between immunoinflammatory biomarkers and CPSP. Regrettably, relevant clinical studies are exceedingly scarce. A single retrospective study evaluated the relationship between CPSP and peripheral blood immunoinflammatory markers in a cohort of 968 individuals following abdominal surgery. This study revealed that preoperative NEU and the changed ratio of NLR were significantly associated with CPSP. Patients in the group with a changed ratio of NLR ≥5 presented a greater incidence of CPSP, an elevated maximum numeric rating scale score post-discharge, an increased prevalence of moderate-to-severe pain, and a more significant impact on quality of life (51). These findings indicate that the care pathways for breast cancer patients should incorporate systematic assessments of immunoinflammatory status and the identification of potential CPSP alongside the implementation of individualized pain management strategies.

Importantly, the subsequent multivariate logistic regression analysis in this study indicated that only BMI independently correlated with CPSP. Although numerous studies have suggested that a high BMI may serve as an independent risk factor for CPSP, considerable high-quality evidence suggests a lack of association between BMI and CPSP, highlighting the complexity of this condition (52–56). Our results indicated that patients with a BMI <22 kg/m² may be at increased risk for developing CPSP. This association may be influenced by the selection criteria for grouping, the limited sample size, and the heterogeneity among studies. In summary, our findings reveal the limited ability of perioperative immunoinflammatory markers to predict CPSP, indicating the influence of additional physiological, psychological, and sociobiological factors on pain outcomes. Therefore, a more comprehensive approach is essential when studying CPSP in breast cancer patients. This approach should evaluate not only immunoinflammatory markers but also physical health, psychological status, and pain history.

While our findings clarify the predictive significance of immunoinflammatory markers for long-term survival and CPSP, it is essential to acknowledge certain limitations. First, the limited sample size may limit the generalizability of our results. Second, the immunoinflammatory biomarker data were obtained from a single center, which may introduce potential biases related to demographic factors such as sex, race, and geographic variations. Finally, patient-specific factors, including preoperative physical condition, psychological status, and perioperative medication regimens, may also impact the incidence of CPSP. Nevertheless, our study offers notable strengths that enhance its contribution to the literature. We conducted regular follow-ups with participants originating from a previous prospective clinical trial over approximately eight years, resulting in reliable data on prognosis outcomes that significantly inform our findings. Moving forward, there is an urgent need for prospective multicenter studies that include larger participant cohorts, comprehensive evaluations of various clinical variables, and more detailed subgroup analysis, especially regarding tumor pathology. Such efforts will deepen our understanding of this promising area of research.

## 5 Conclusion

In summary, this study highlights the predictive significance of preoperative immunoinflammatory biomarkers for long-term survival in patients who underwent breast cancer surgery. The finding that an elevated preoperative SIRI serves as an independent risk factor for DFS emphasizes the necessity of integrating inflammatory evaluation into clinical practice. The correlation between perioperative immunoinflammatory biomarkers and CPSP warrants further investigation to elucidate the intricacies of postoperative pain. Integrating a comprehensive understanding of inflammation, tumors, and pain will help to enhance individualized medical strategies, resulting in improved outcomes and quality of life for breast cancer patients.

## Data Availability

The original contributions presented in the study are included in the article/**Supplementary Material**. Further inquiries can be directed to the corresponding authors.
