# Supplementary material for: Peripheral blood immunoinflammatory biomarkers: prospective predictors of postoperative long-term survival and chronic postsurgical pain in breast cancer
Source: Front Immunol. 2025 Jan 29;16:1531639. doi: 10.3389/fimmu.2025.1531639 (PMC11813937; doi:10.3389/fimmu.2025.1531639)
Supplement: Supplementary file 1 [file DataSheet1.pdf]

Peripheral Blood Immunoinflammatory Biomarkers: Prospective Predictors of Postoperative Long-term Survival and Chronic Postsurgical Pain in Breast Cancer

Supplementary Material

1 Supplementary Figures

Supplementary Figure 1 Kaplan–Meier curves and log-rank tests for the relationships between clinicopathological characteristics and the long-term survival. **A1-A2:** Kaplan–Meier curves for DFS and OS by age; **B1-B2:** Kaplan–Meier curves for DFS and OS by BMI; **C1-C2:** Kaplan–Meier curves for DFS and OS by menopausal; **D1-D2:** Kaplan–Meier curves for DFS and OS by tumor size; **E1-E2:** Kaplan–Meier curves for DFS and OS by tumor type; **F1-F2:** Kaplan–Meier curves for DFS and OS by carcinoma cell embolus; **G1-G2:** Kaplan–Meier curves for DFS and OS by nerve infiltration; **H1-H2:** Kaplan–Meier curves for DFS and OS by lymph node metastasis; **I1-I2:** Kaplan–Meier curves for DFS and OS by TNM stage; **J1-J2:** Kaplan–Meier curves for DFS and OS by ER; **K1-K2:** Kaplan–Meier curves for DFS and OS by PR; **L1-L2:** Kaplan–Meier curves for DFS and OS by HER2; **M1-M2:** Kaplan–Meier curves for DFS and OS by Ki-67; **N1-N2:** Kaplan–Meier curves for DFS and OS by histological grade; **O1-O2:** Kaplan–Meier curves for DFS and OS by postoperative chemotherapy; **P1-P2:** Kaplan–Meier curves for DFS and OS by postoperative radiotherapy; **Q1-Q2:** Kaplan–Meier curves for DFS and OS by adjuvant endocrine therapy; **R1-R2:** Kaplan–Meier curves for DFS and OS by targeted therapy. BMI, body mass index; TNM, tumor node metastasis; ER, estrogen receptor; PR, progesterone receptor; HER2, human epidermal growth factor receptor 2; DFS, disease-free survival; OS, overall survival.

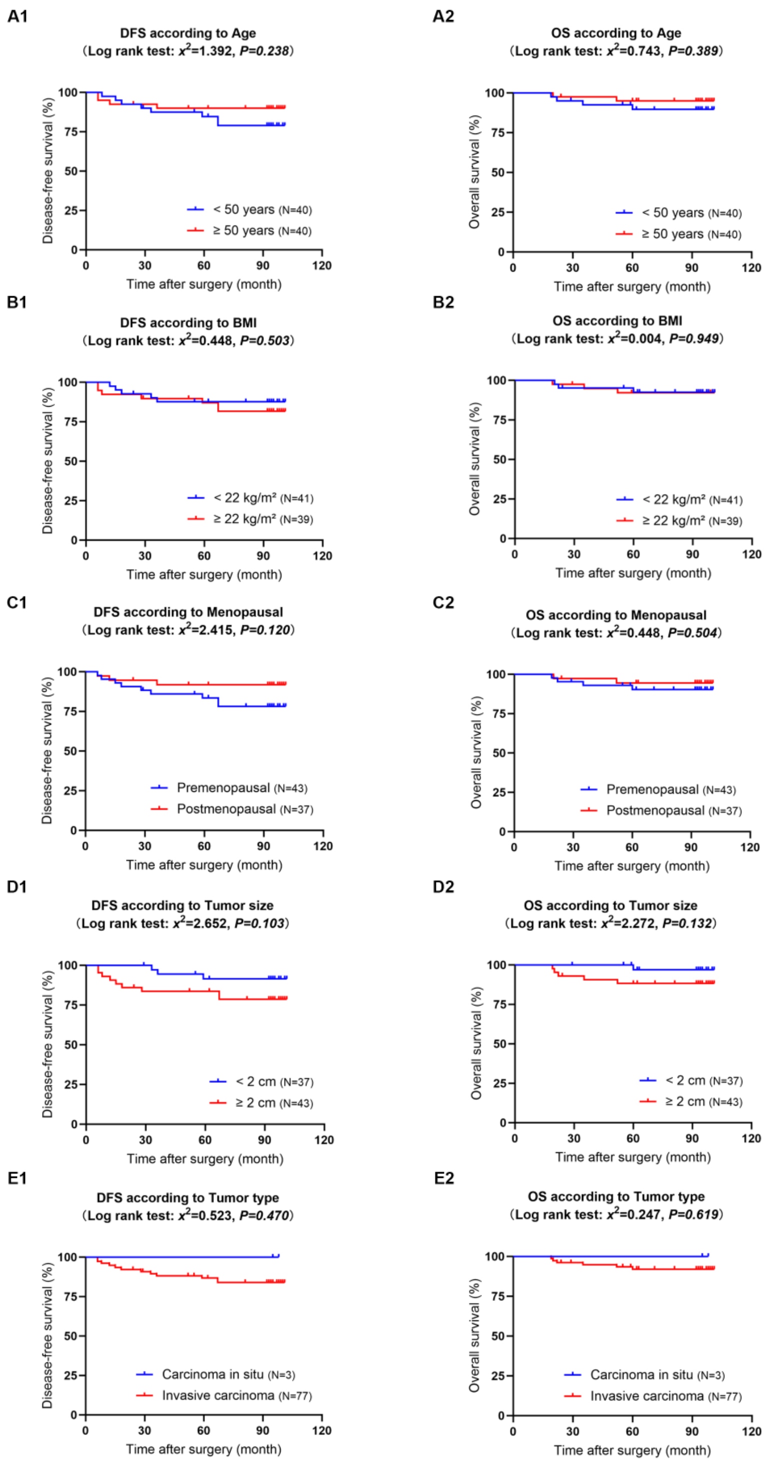

Supplementary Figure 1 Kaplan–Meier curves and log-rank tests for the relationships between clinicopathological characteristics and the long-term survival.

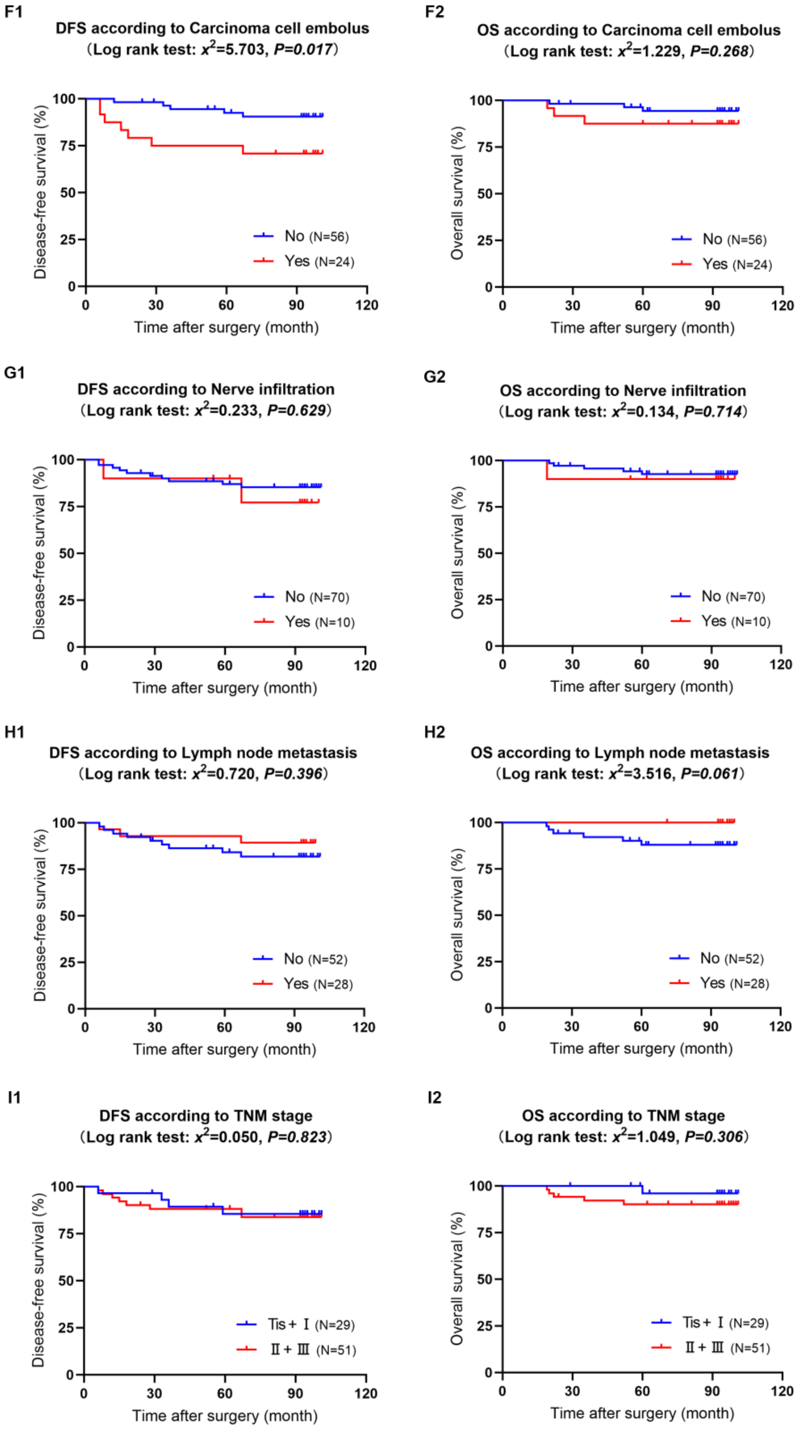

Supplementary Figure 1 (continued) Kaplan–Meier curves and log-rank tests for the relationships between clinicopathological characteristics and the long-term survival.

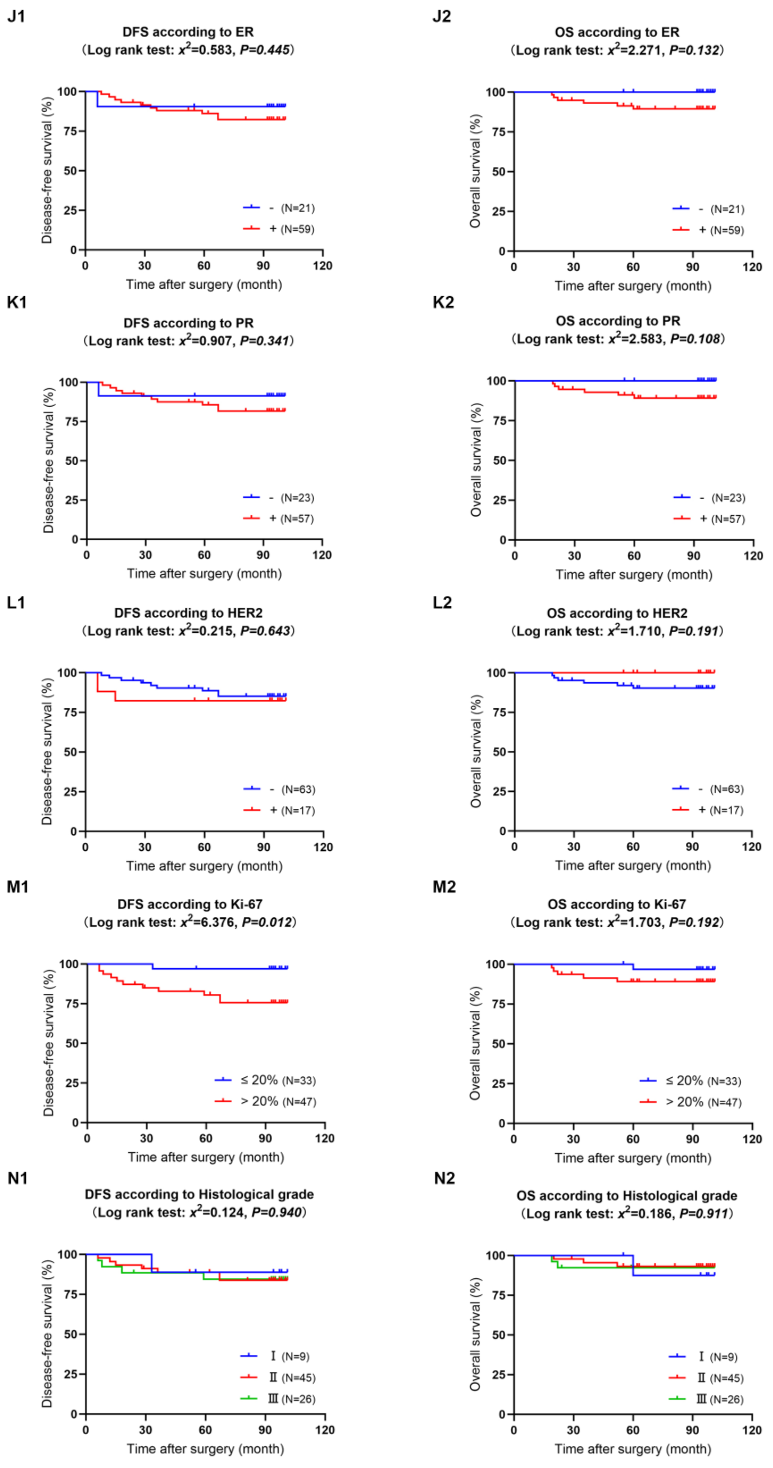

Supplementary Figure 1 (continued) Kaplan–Meier curves and log-rank tests for the relationships between clinicopathological characteristics and the long-term survival.

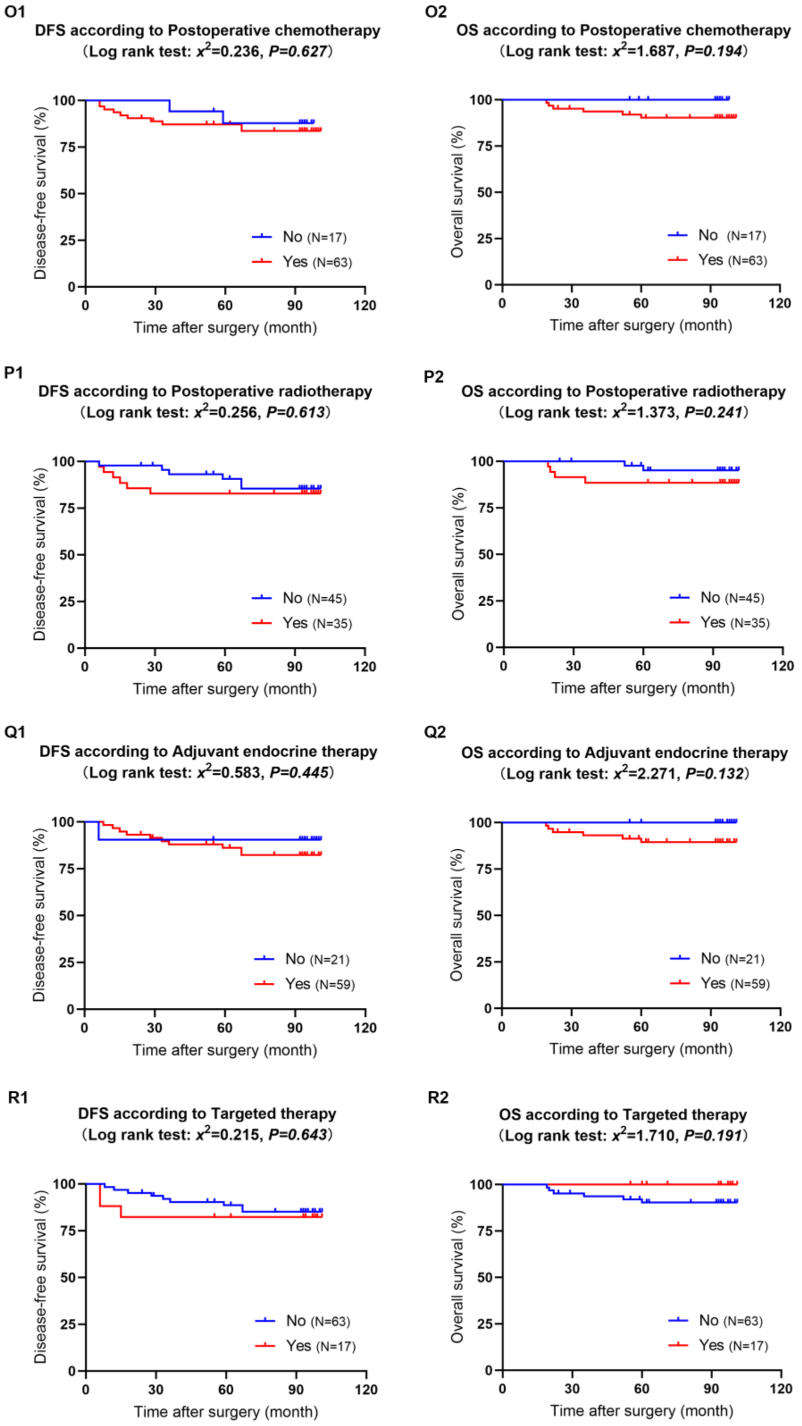

Supplementary Figure 1 (continued) Kaplan–Meier curves and log-rank tests for the relationships between clinicopathological characteristics and the long-term survival.

2 Supplementary Tables

Supplementary Table 1 Comparison of dynamic changes in peripheral blood cell counts between the CPSP group and the non-CPSP group.

Characteristic	CPSP (N=23)	Non-CPSP (N=57)	*P*
**Difference in NEU** (post NEU - pre NEU, $\times 10^9$/L)	2.62(2.15,3.25)	3.39(2.31,5.01)	**0.026**
**Difference in MONO** (post MONO - pre MONO, $\times 10^9$/L)	0.17(0.07,0.24)	0.21(0.14,0.30)	0.101
**Difference in LYM** (post LYM - pre LYM, $\times 10^9$/L)	-0.92(-1.50,-0.52)	-0.83(-1.39,-0.52)	0.894
**Difference in PLT** (post PLT - pre PLT, $\times 10^9$/L)	-9.00(-29.00,3.00)	-8.00(-29.00,5.00)	0.996
**Ratio of NEU** (post NEU/pre NEU)	1.73(1.54,1.93)	1.98(1.67,2.53)	0.062
**Ratio of MONO** (post MONO/pre MONO)	1.44(1.20,1.79)	1.57(1.31,1.86)	0.350
**Ratio of LYM** (post LYM/pre LYM)	0.49(0.35,0.64)	0.49(0.39,0.63)	0.844
**Ratio of PLT** (post PLT/pre PLT)	0.96(0.88,1.01)	0.97(0.88,1.01)	0.861

CPSP, chronic postsurgical pain; pre, preoperative; post, postoperative; NEU, neutrophil count; MONO, monocyte count; LYM, lymphocyte count; PLT, platelet count. Continuous variables are summarized as the means ± SDs or medians (P25, P75). Categorical variables are summarized as the number of subjects and percentage. The values in bold represent P <0.05.

Supplementary Table 2 Univariate logistic regression analysis of CPSP according to dynamic changes in peripheral blood cell counts.

Characteristic	OR	95% CI	*P*
**Difference in NEU** (post NEU - pre NEU, $\times 10^9$/L)	0.779	0.606-1.001	0.051
**Difference in MONO** (post MONO - pre MONO, $\times 10^9$/L)	0.268	0.044-1.623	0.152
**Difference in LYM** (post LYM - pre LYM, $\times 10^9$/L)	1.084	0.505-2.328	0.836
**Difference in PLT** (post PLT - pre PLT, $\times 10^9$/L)	0.999	0.988-1.010	0.853
**Ratio of NEU** (post NEU/pre NEU)	0.575	0.273-1.210	0.145
**Ratio of MONO** (post MONO/pre MONO)	0.605	0.246-1.486	0.273
**Ratio of LYM** (post LYM/pre LYM)	0.971	0.138-6.806	0.976
**Ratio of PLT** (post PLT/pre PLT)	0.542	0.057-5.150	0.594

CPSP, chronic postsurgical pain; OR, odds ratio; CI, confidence interval; pre, preoperative; post, postoperative; NEU, neutrophil count; MONO, monocyte count; LYM, lymphocyte count; PLT, platelet count. The values in bold represent P <0.05.
